# Privacy Protection in Online Health Communities: Natural Experimental Empirical Study

**DOI:** 10.2196/16246

**Published:** 2020-05-21

**Authors:** Yuanyuan Dang, Shanshan Guo, Xitong Guo, Doug Vogel

**Affiliations:** 1 School of Management Harbin Institute of Technology Harbin China

**Keywords:** online health community, privacy protection, professional health care knowledge

## Abstract

**Background:**

An online health community (OHC) is a novel sharing channel through which doctors share professional health care knowledge with patients. While doctors have the authority to protect their patients’ privacy in OHCs, we have limited information on how doctors’ privacy protection choices affect their professional health care knowledge sharing with patients.

**Objective:**

We examined the relationship between privacy protection and professional health care knowledge sharing in OHCs. Specifically, we examined the effects of privacy protection settings in an OHC on doctors’ interactive professional health care knowledge sharing and searching professional health care knowledge sharing (two dimensions of professional health care knowledge sharing). Moreover, we explored how such effects differ across different levels of disease stigma.

**Methods:**

We collected the monthly panel data of 19,456 doctors from Good Doctor, one of the largest OHCs in China, from January 2008 to April 2016. A natural experimental empirical study with difference-in-difference analysis was conducted to test our hypotheses. The time fixed effect and the individual fixed effect were both considered to better identify the effects of a privacy protection setting on professional health care knowledge sharing. Additionally, a cross-sectional analysis was performed for a robust check.

**Results:**

The results indicate that the privacy protection setting has a significant positive effect on interactive professional health care knowledge sharing (β=.123, *P*<.001). However, the privacy protection setting has a significant negative effect on searching professional health care knowledge sharing (β=–.225, *P*=.05). Moreover, we found that high disease stigma positively impacts the effect of privacy protection on interactive professional health care knowledge sharing (coefficients are in the same valence) and negatively impacts the effects of privacy protection on searching professional health care knowledge sharing (coefficients are in the reverse valence).

**Conclusions:**

Privacy protection has a bilateral effect on professional health care knowledge sharing (ie, a positive effect on interactive professional health care knowledge sharing and a negative effect on searching professional health care knowledge sharing). Such bilateral switches of professional health care knowledge sharing call for a balanced state in conjunction with practical implications. This research also identifies a moderate effect of disease stigma on privacy protection settings and professional health care knowledge sharing.

## Introduction

### Online Health Communities as Platforms for Sharing Professional Health Care Knowledge

The internet has dynamically invaded, affected, and even changed many traditional industries, such as business (electronic commerce [e-commerce]), finance (internet finance), social (virtual communities), and health care (telemedicine). Compared with the traditional offline mode, the capabilities of the internet (ie, its real-time communication across geographical boundaries, wide and swift spread, easy access, and rapid generation of users) have proven successful in the online mode. Recently, studies in both practice and research have focused on another internet capability: sharing, especially in the sharing economy. For instance, travel apps help in sharing transportation tools (eg, Uber), cloud computing apps help in sharing idle computing capacities (eg, Alibaba Cloud computing), and accommodation apps help in sharing lodgings (eg, Airbnb). In addition, regarding the health care industry, the online health community (OHC) (a kind of telemedicine-providing, doctor-patient means of communication using information and communication technology [1]) provides an online platform for sharing professional health care knowledge.

Similarly, with transportation tools, computing capacities, and lodgings, professional health care knowledge is a scarce and valuable resource. Having abundant and correct professional health care knowledge is conducive to enhancing coping and self-efficacy [2], affecting health-related decisions and the behavior of OHC users and their friends and family [3], enabling better management of chronic health conditions [4], and fueling discussions with health care providers [5]; health care, in general, is thereby maintained. However, most professional health care knowledge networks are owned by a small number of health care professionals. In traditional offline health care activities, professional health care knowledge is mainly shared through face-to-face treatment. In particular, it makes for inefficient sharing in a one-to-one context.

According to the definition of knowledge sharing [6,7], professional health care knowledge sharing is defined as medical professionals (eg, doctors, nurses, and pharmacists) sharing their professional health care knowledge with other health care stakeholders in the form of various activities (eg, treatment, consultations, and published papers) [8]. Fortunately, the OHC provides more possibilities for professional health care knowledge sharing, including the seeking of advice online and by telephone consultation [9,10]; for connecting with other patients with similar experiences [11,12]; and for the understanding of professional diagnoses by reading doctors’ published papers. Moreover, the OHC supplies to the public the online interaction records between doctors and patients to help patients with similar ailments [13,14]. From the perspective of doctors, the professional health care knowledge sharing between doctors and patients can be divided into interactive professional health care knowledge sharing and searching professional health care knowledge sharing. Interactive professional health care knowledge sharing refers to the professional health care knowledge sharing process actively carried out by doctors through interaction with patients, such as online consultations and telephone consultations in OHCs. Searching professional health care knowledge sharing, on the other hand, refers to a process where there are no doctor-patient interactions; patients search, learn, and acquire doctors’ professional health care knowledge autonomously, such as by searching doctors’ published articles and doctor-patient interaction records (in Chinese OHCs, such doctor-patient interaction records, which are anonymously processed, are on display to all visitors by default, like those in the forums). Professional health care knowledge sharing, based on the internet, is being adopted by a growing number of users [15].

### Privacy Protection and Professional Health Care Knowledge Sharing in OHCs

Unlike transportation tools, computing capacities, and lodgings, professional health care knowledge sharing is more inclined to be privacy-sensitive. It is more sensitive than other forms of information on online platforms, such as demographic proﬁles, lifestyle interests, or purchase history information [16]. Just as in an offline context, doctors make a diagnosis according to a patient’s conditions; therefore, professionals may ask their patients to provide more personal data in exchange for the utility of the services. To obtain better treatment, manage their conditions, or improve their overall health, patients have to provide their personal information (such as gender, lifestyle, occupation, disease severity, and medical examination results), which may contain more sensitive personal information [17]. Moreover, professionals also post sensitive data about their patients, which is made visible to the public, so that advice on clinical situations or practice management may be shared with other patients or professionals [18,19].

OHCs are opening the door for inappropriate access, misuse, and disclosure of personal privacy [20]. Once such data are handled improperly, patients will be faced with privacy invasion [21], such as negative social stigma, as well as straining of family ties, losing control of medical information, and suffering harassment from commercial advertising [16]. These concerns can even cause individuals to avoid the health care services of OHCs, and thus limit the role of an OHC in professional health care knowledge sharing. Consequently, some OHCs have provided privacy protection mechanisms, such as improving data storage security, building a safe environment, and providing anonymity for patients. In addition, an OHC can enable doctors to activate their patients’ privacy protection settings by making the consultation process invisible.

### Objectives and Research Questions

Several studies (further previous research can be seen in Multimedia Appendix 1) have investigated professional health care knowledge sharing in OHCs among patients or doctors, such as the patient motivations for health care knowledge sharing on Wikipedia [22] and the development of doctors’ knowledge sharing in virtual communities [23,24]. However, a dearth of studies concentrating on professional health care knowledge sharing between doctors and patients remains [25]. Moreover, although studies have explored the positive impact of privacy protection mechanisms on patients’ OHC participation intentions and continuous use intentions [16,21], privacy protection is also a double-edged sword [26] for professional health care knowledge sharing. Doctors’ privacy protection settings in OHCs regarding patients’ personal health care information will increase patient trust and promote knowledge sharing. However, it also blocks other patients’ access to professional health care knowledge in their search for consultation records. Therefore, it is necessary to explore the influence of doctors’ choice of privacy protection settings in OHCs on professional health care knowledge sharing between doctors and patients. To fill this research gap, it is essential to understand the nature of professional health care knowledge sharing in OHCs. Hence, we conducted an empirical analysis of a large OHC to explore the effects of privacy protection settings on interactive and searching professional health care knowledge sharing. In further extending our model, we investigated how professional health care knowledge is differentially shared with patients suffering from different levels of disease stigma. For instance, patients with different diseases (eg, influenza versus HIV) may have different professional health care knowledge sharing trajectories from the influence of the associated stigma.

### The Enhancing Power of Privacy Protection on Interactive Professional Health Care Knowledge Sharing

In OHCs, interactive forms of professional health care knowledge sharing are mainly conducted through online doctor-patient consultations. In other words, an online consultation thread can be started for a doctor-and-patient pair, and each can interact with the other through text descriptions and the uploading of test and imaging results. Doctors can make judgments and recommendations based on their patients’ descriptions of their symptoms to generate professional health care knowledge sharing in their interactions with patients. Without privacy protection, the entire online consultation process described above would be rendered publicly available to all internet users. However, as we mentioned previously, this may be harmful in terms of patients’ privacy disclosure. The privacy protection mechanism of an OHC gives doctors the right to set up a transparent consultation process, so as to protect the privacy of their patients.

Previous studies indicated that a privacy policy can improve user trust and loyalty in health care providers, thus influencing health information exchange behaviors in users [27]. In the interactive professional health care knowledge sharing process of an OHC, patients may increase their trust in doctors when they are protected by doctors’ privacy protection settings, thus feeling encouraged to share more personal health care information during communication [28]. The feeling of being protected will also increase patients’ evaluations of doctors and render them willing to communicate with doctors more frequently, thus increasing the shared professional health care knowledge in the interactive process. Further, doctors’ reasonable privacy protection settings will attract more patients to consult online for professional health care knowledge sharing. Hence, by increasing patient interaction intentions, consultation times, and the number of patients, doctors’ privacy protection settings can affect the professional health care knowledge sharing frequency of the doctor-patient interaction process.

Thus, we hypothesized the following:

H1: Privacy protection settings have a positive impact on a doctor’s interactive professional health care knowledge sharing in OHCs.

### Conflicts Between Privacy Protection and Searching Professional Health Care Knowledge Sharing

In addition to interactive professional health care knowledge sharing, searching professional health care knowledge sharing is another approach by which doctors can share knowledge in OHCs. As there are numerous visible consultation records in OHCs that are available to other users, it is easy for a patient to access these consultation records via search engines (eg, Bing, Google, or Yahoo!) [29]. By learning from the professional health care knowledge that doctors share with other patients in their records, patients can internalize such knowledge as their professional health care knowledge.

For doctors, searching professional health care knowledge sharing is a more widely used method of disseminating professional knowledge than interactive professional health care knowledge sharing. Compared with interactive professional health care knowledge sharing in one-to-one communication, searching professional health care knowledge can be extended to any patient who is willing to share such knowledge through the internet under the authorization of doctors. Thus, searching professional health care knowledge sharing provides a more convenient knowledge sharing channel for doctors. According to statistics, among the patients who use the internet to obtain professional health care knowledge, 61% use search engines to access searching professional health care knowledge sharing, which attracts twice the number of users who use social platforms and online communities [3].

Searching professional health care knowledge sharing, which is mainly recorded in doctor-patient interactive history records, often contains patients’ specific symptoms, diagnosis results, examination records, and other matters protected by personal privacy. Although these history records will help other patients acquire searching professional health care knowledge sharing more accurately, if such private information is used indiscreetly, it will have adverse effects on those whose privacy has been invaded [16]. Therefore, the OHC provides a function for doctors to protect the privacy of their patients by making the consultation process invisible. It helps to eliminate the privacy exposure of personal health care information and thus fundamentally eliminates privacy invasion. However, while privacy is protected, other patients are not able to access the complete interaction records and, thus, are not able to clearly grasp the details of the shared professional health care knowledge. This negatively affects the searching professional health care knowledge sharing of other patients.

Thus, we hypothesized the following:

H2: Privacy protection settings have a negative impact on a doctor’s searching professional health care knowledge sharing in OHCs.

### The Moderating Effects of Disease Stigma

Closely related to privacy protection in OHCs is the inherent stigma ascribed to many health conditions [30]. Social stigma refers to negative feelings toward an individual or a group on socially characteristic grounds that distinguish the individual or group from others [31,32].

Compared to offline treatments, online professional health care knowledge sharing provides natural protection, like a veil, for patients with diseases they may feel embarrassed about. Through an OHC, patients with stigma disorders can access professional health care knowledge sharing with doctors without the need for face-to-face interactions. In particular, people living with stigmatized conditions such as mental illness, cancer, or HIV are more likely to seek support and health information online due to the perceived anonymity the internet provides [33-35]. In their study, Zhang et al [16] demonstrated that the internet could support the disclosure of stigmatized illnesses such as HIV by helping the afflicted overcome the aspects of social stigma.

Different types of diseases possess different degrees of stigma. For instance, patients with HIV will be more worried about and feel more ashamed of other people’s discrimination than patients with a cold. Berger et al [35] showed that compared to those with nonstigmatized diseases, those with stigmatized illnesses were more likely to be sensitive to privacy disclosure. This is because once a stigmatized patient’s privacy is exposed, the disclosure will cause the patient more considerable pain and annoyance than for a nonstigmatized patient. Therefore, when patients with stigmatized diseases use OHCs to interact with doctors to search for professional health care knowledge sharing, they expect their privacy to be fully protected. Patients with stigmatized diseases, who are more sensitive about protecting their privacy, are more likely to trust doctors who devote attention to protecting their patients’ privacy. As a consequence, their frequency of searching professional health care knowledge sharing increases.

Thus, we hypothesized the following:

H3: The stigma associated with a disease positively moderates the effects of privacy protection settings on interactive and searching professional health care knowledge sharing.

## Methods

### Research Settings

One of the biggest OHCs in China, Good Doctor (http://www.haodf.com/), which was launched in September 2006, was chosen for our research context. It provides a virtual online platform for patients and offline registered doctors. Through Good Doctor, patients can engage in professional health care knowledge sharing with doctors through online consultations, telephone consultations, reading articles published by doctors, browsing other patient-doctor consultation records, and so on. These professional health care knowledge sharing processes may be recorded by Good Doctor, and some of them are shown to the public on doctors’ profile pages. The professional health care knowledge sharing interaction records include telephone and online consultation records (ie, clinical and academic titles and detailed content of the consultation, from the doctor and patient, during the whole consultation process) with anonymous patients, the number and content of articles published by the doctor (ie, academic papers, summary of medical experience, and related knowledge forwarding), and the total number of patients who have engaged in professional health care knowledge sharing with the doctor. However, if the doctor has set privacy protection on an online consultation, the consultation details are not rendered publicly available. We developed software that crawled doctors’ profile pages in Good Doctor every month from January 2008 to April 2016. Finally, we constructed a monthly panel data set for 19,456 doctors.

### Variables

Professional health care knowledge sharing was measured by the number of patients who interacted with a doctor or searched the consultation records of that doctor. The number of online consultations between patients and a doctor through which they engage in professional health care knowledge sharing interaction was counted based on the doctor’s consultation list and was used to measure our first dependent variable: the doctor’s interactive professional health care knowledge sharing (intrctPHKS). The second dependent variable, searching professional health care knowledge sharing (searchPHKS), indicates the number of patients who learned of a doctor’s professional health care knowledge through searching that doctor’s consultation records without interacting with the doctor. Although this number cannot be collected directly from the website, it can be inferred from the calculation of the number of patients who visit the doctor’s profile page minus the number of patients who interact via consultations. Even though the number of patients visiting the profile page may count in “noise” values (such as browsing a doctor’s personal information, or repeat visits), in the difference-in-difference model, a random error in the dependent variable will not influence regression estimates of treatment effect. Hence, by ignoring the cases of a patient viewing a doctor’s profile page unintentionally, it is assumed that all patients visiting a doctor’s page have access to professional health care knowledge sharing with this doctor. In other words, in addition to the number of patients interacting with a doctor through consultations, patients who visit a doctor’s page have access to searching professional health care knowledge sharing with that doctor.

Online consultations between doctors and patients can be conducted by means of text and images, and the records of consultations are open to the public unless the doctor changes the privacy protection settings of the records. During a doctor-patient consultation, the doctor has the authority to choose whether to make the consultation records of their patient visible to the public. If an online consultation is set as private, only the patient and the attending doctor can access the entire interaction history. Others who visit this doctor’s profile page can view only the topic of a private consultation without seeing the detailed information. We calculated both the total number of a doctor’s online consultations with privacy protection settings and the monthly number of privacy protection settings to measure our independent variable: PrivacySetting.

Moreover, patients with different levels of disease stigma may have different attitudes toward privacy sensitivity regarding online professional health care knowledge sharing interactions with doctors. To understand how the stigma of diseases affects the privacy protection setting of consultations on interactive and searching professional health care knowledge sharing, disease stigma (Stigma) was considered as the moderate effect of our model. We used the classification method of De Choudhury et al [8] to measure stigma on a 2-point scale: 1=high stigma and 0=low stigma.

To account for potential confounding effects that influence professional health care knowledge sharing, we included other control variables (ie, the doctor’s title, virtual gifts, articles posted online, and thank-you letters). According to the literature, a doctor’s offline status and online reputation will also affect their knowledge sharing in OHCs [36]. Therefore, the variables that represent the doctor’s offline status and online reputation are treated as our control variables (ie, the title of a doctor [Title], the number of virtual gifts [Gift], the number of thank-you letters [Letter], and the number of published articles [Article]). The official clinical title certified by China’s national agency based on uniform standards was used to measure Title. In general, 4 rankings exist for titles: Fellow (4), Associate Fellow (3), Attending (2), Resident (1), and none (0). The variable Gift was used to represent the number of virtual gifts sent by the patients who consulted online. Thank-you letters (Letter) denote the number of thank-you letters submitted by patients who had visited a doctor in both the online and offline contexts. The number of articles the doctor had published online was counted as Article. The defined variables are shown in Table 1.

### Descriptive Statistics

Our monthly panel data set included the doctor’s identification number, the number of online consultations with and without privacy protection settings, the total number of visits, the number of patients, the number of articles, the number of gifts, the number of follow-up visits, and the title of the doctor. We obtained 631,529 online consultations of 19,456 doctors. More detailed descriptive statistics are shown in Table 2.

The correlation of each indicator using a cross-section is shown in Table 3, which indicates values in the acceptable range. The correlation coefficients between the independent variables are relatively small. In other words, the possibility of variable redundancy is relatively small.

**Table 1:** Variables defined.

Variable		Operational definition
**Dependent**		
	intrctPHKS^a^	The number of interactive professional health care knowledge sharing
	searchPHKS^b^	The number of searching professional health care knowledge sharing
**Independent**		
	PrivacySetting	The number of online consultations with a privacy protection setting
**Moderate**		
	Stigma	The stigma of a disease for which a doctor offers consultations
**Control**		
	Gift	The number of virtual gifts received by a doctor
	Letter	The number of thank-you letters
	Title	The title of the doctor
	Article	The number of articles written by the doctor

^a^intrctPHKS: interactive professional health care knowledge sharing.

^b^searchPHKS: searching professional health care knowledge sharing.

**Table 2:** Descriptive statistics for selected variables.

Variable		Observed	Mean (SD)	Minimum	Maximum
**Dependent**					
	intrctPHKS^a^	19,456	459.9 (1528)	0	64,561
	searchPHKS^b^	19,456	333,510 (1,420,069)	7	62,300,000
**Independent**					
	PrivacySetting	19,456	0.133 (1.106)	0	23
**Moderate**					
	Stigma	19,456	0.403 (0.491)	0	1
**Control**					
	Gift	19,456	20.67 (96.87)	0	2996
	Letter	19,456	6.723 (19.26)	0	382
	Title	19,456	3.895 (0.923)	1	5
	Article	19,456	8.920 (46.82)	0	2158

^a^intrctPHKS: interactive professional health care knowledge sharing.

^b^searchPHKS: searching professional health care knowledge sharing.

**Table 3:** Correlation coefficients between independent variables.

Variable	intrctPHKS^a^	searchPHKS^b^	PrivacySetting	Stigma	Gift	Letter	Title	Article
intrctPHKS	1.000	—^c^	—	—	—	—	—	—
searchPHKS	0.904	1.000	—	—	—	—	—	—
PrivacySetting	0.097	0.099	1.000	—	—	—	—	—
Stigma	0.016	0.006	0.014	1.000	—	—	—	—
Gift	0.733	0.688	0.081	0.034	1.000	—	—	—
Letter	0.676	0.606	0.086	0.050	0.704	1.000	—	—
Title	0.141	0.130	0.011	0.110	0.115	0.202	1.000	—
Article	0.302	0.366	0.051	0.039	0.211	0.184	0.074	1.000

^a^intrctPHKS: interactive professional health care knowledge sharing.

^b^searchPHKS: searching professional health care knowledge sharing.

^c^N/A: not applicable.

### Empirical Models

Observing the privacy protection setting of doctors over various periods creates a natural experimental setting that allows a comparison of the effects of differences in privacy protection settings before and after a doctor uses this function. Constructing a panel data set of doctors’ monthly online consultations, we estimated the difference-in-difference models, reflected by equations (1) through (4). Difference-in-difference models were used to calculate the effect of a treatment on an outcome by comparing the outcome average change over time from the control group to the treatment group. It helps to remove biases of permanent differences between the treatment and control groups, as well as trend biases caused by the changing of other factors of the outcome over time [37]. Thus, we exploited the effect of privacy protection settings on professional health care knowledge sharing by identifying an exogenous variation of professional health care knowledge sharing that the privacy protection settings are set by different doctors in different months. This identification strategy has been implemented in several extant studies (eg, in Chan and Ghose [38]). Our estimation incorporated doctor-level fixed effects, which allowed us to effectively control for doctor-level unobserved heterogeneity. To control the trend effect of each doctor’s professional health care knowledge sharing, we added the time fixed effect in all 4 models. Moreover, we added the individual fixed effect to control the omitted variable bias caused by unobserved heterogeneity. Both the time fixed effect and the individual fixed effect were set as dummy variables. Furthermore, we used a cross-sectional data set to conclude our analyses with a set of robustness checks. Accordingly, our empirical models of doctors’ privacy protection settings in OHCs on both their interactive and searching professional health care knowledge sharing are as follows:

intrctPHKS_it_ = β_0_ + β_1_ PrivacySetting_it_ + β_2_ Stigma_i_ + β_3_C + A_i_ + T_t_ + ε_it_ (1)

searchPHKS_it_ = β_0_ + β_1_ PrivacySetting_it_ + β_2_ Stigma_i_ + β_3_C + A_i_ + T_t_ + ε_it_ (2)

The moderate effect models are as follows:

intrctPHKS_it_ = β_0_ + β_1_ PrivacySetting_it_ + β_2_ Stigma_i_ + β_7_ Stigma_it_*PrivacySetting_it_ + β_3_C + A_i_ + T_t_ + ε_it_ (3)

searchPHKS_it_ = β_0_ + β_1_ PrivacySetting_it_ + β_2_ Stigma_i_ + β_7_ Stigma_it_*PrivacySetting_it_ + β_3_C + A_i_ + T_t_ + ε_it_ (4)

Where *i* indexes indicate the doctor and *t* indexes indicate time (monthly). *A_i_* is a vector of doctor fixed effects; *T_t_* is a vector of time fixed effects; *PrivacySetting_it_* is the binary indicator for the privacy protection setting (that is, PrivacySetting_it_=1 if the doctor has set the privacy in a particular month; zero otherwise); and *ε_it_* is an error term. The coefficient *β*_1_ is the difference-in-difference estimate of the effect of a privacy protection setting. If β_1_>0, then the privacy protection setting has caused an increase in professional health care knowledge sharing. Control variables are indicated by *C*.

## Results

### Hypothesis Testing

Stata (StataCorp) was used for our statistical analysis, and the results are shown in Table 4. The adjusted R^2^ (0.037, –0.195, 0.037, and –0.223 for models 1, 2, 3, and 4 respectively) and *F* values (*F*_101,143415_=384.8419, *F*_102,143414_=381.0676, *F*_101,143376_=34.43728, and *F*_3,143474_=5.534647 for models 1, 2, 3, and 4 respectively) were reasonable and significant. The results of the variance inflation factor statistics for the variable indicated no multicollinearity (the variance inflation factor statistic of every variable is not greater than 2.0). The results of models only with our main treatment effect (PrivacySetting) are shown in columns 1 and 3 for interactive professional health care knowledge sharing and searching professional health care knowledge sharing, respectively. The results of models added to the moderate effects of stigma are shown in columns 2 and 4 for interactive professional health care knowledge sharing and searching professional health care knowledge sharing, respectively.

Hypothesis 1 postulated that the privacy protection setting has a significant positive effect on interactive professional health care knowledge sharing. In column 1 of Table 4, this hypothesis is supported as the coefficient of privacy protection setting was seen as positive and statistically significant (β_1_=.123, *P*<.001). However, we observed in column 3 a significantly negative effect of the privacy protection setting on doctors’ searching professional health care knowledge sharing (β_1_=–.225, *P*=.050). Therefore, hypothesis 2, which posits that the privacy protection setting has a negative effect on searching professional health care knowledge sharing online, is also supported. As both the dependent variables of model 1 (intrctPHKS) and model 3 (searchPHKS) represent the amount of a doctor’s knowledge sharing, a Wald test [39] was applied to the coefficients for further comparison. In the results for PrivacySetting, the absolute value of the coefficient of searching professional health care knowledge sharing was significantly larger than that of interactive professional health care knowledge sharing (Wald test, *F*_2,175548_=324.21; *P*<.001).

Hypothesis 3 investigated the moderate impact of disease stigma on the relation between privacy protection settings and professional health care knowledge sharing. Columns 2 and 4 of Table 4 show the stable significant result of the positive moderate effect of disease stigma. In other words, high disease stigma positively impacts the effects of privacy protection settings on interactive professional health care knowledge sharing (coefficients are in the same valence) and negatively impacts the effects of privacy protection settings on searching professional health care knowledge sharing (coefficients are in the reverse valence). Therefore, hypothesis 3 is verified. Specifically, for disease stigma, we found that the stigma has a positive correlation with professional health care knowledge sharing.

### Robust Check

To check the robustness of our results, we conducted a cross-sectional rerun of the model. The results presented in Table 5 are consistent with the results of the previous model. The results show that the treatment and the moderate effect have a significant positive effect, which is the same as the main results.

All the control variables have a statistically significant effect on professional health care knowledge sharing. Doctors with higher online and offline reputations will attract more patients [40]. The title reflects a doctor’s offline reputation, and higher-level doctors are known to be more professional. Hence, the title has a positive effect on doctors’ professional health care knowledge sharing. Moreover, virtual gifts, the number of articles, and thank-you letters can be proxy variables of a doctor’s online reputation, which can positively affect professional health care knowledge sharing.

**Table 4:** Econometric models of the treatment effect and moderate effect. All the control variables and the individual and time fixed effect variables were included in the 4 models. The interaction terms (PrivacySetting*Stigma) represent the moderate effect of stigma.

Variable	intrctPHKS^a^ (N=175,548)				searchPHKS^b^ (N=175,509)			
	Model 1		Model 2		Model 3		Model 4	
	Treatment effect		Moderate effect		Treatment effect		Moderate effect	
	β (SE)^c^	*P* value	β (SE)	*P* value	β (SE)	*P* value	β (SE)	*P* value
PrivacySetting	.123 (.063)	<.001	.105 (.084)	.009	–.225 (.024)	.05	–.224 (.032)	<.001
Stigma	.024 (.005)	<.001	.024 (.005)	<.001	.033 (.005)	.008	.044 (.005)	.003
PrivacySetting*Stigma	—^d^	—	.041 (.125)	.08	—	—	.089 (.049)	.07
Constant	2.665 (3.482)	.44	2.665 (3.482)	.44	.414 (1.335)	.76	.376 (.003)	.001
Control	YES	N/A^e^	YES	N/A	YES	N/A	YES	N/A
Individual fixed effect	YES	N/A	YES	N/A	YES	N/A	YES	N/A
Time fixed effect	YES	N/A	YES	N/A	YES	N/A	YES	N/A
*R* ^2^	.037	N/A	.037	N/A	–0.195	N/A	–.223	N/A

^a^intrctPHKS: interactive professional health care knowledge sharing.

^b^searchPHKS: searching professional health care knowledge sharing.

^c^Values in parentheses are the robust standard errors.

^d^Not available. Models 1 and 3 did not contain the moderate effect of stigma.

^e^N/A: not applicable.

**Table 5:** Results of the robust check using cross-section data (N=23,112 for all models).

Variable	intrctPHKS^a^				searchPHKS^b^			
	Model 1		Model 2		Model 3		Model 4	
	β (SE)^c^	*P* value	β (SE)	*P* value	β (SE)	*P* value	β (SE)	*P* value
PrivacySetting	.0196 (4.35)	<.001	.0277 (4.71)	<.001	–.030 (.012)	.05	–.032 (.013)	.02
Stigma	.0308 (6.86)	<.001	.0297 (6.55)	<.001	.032 (0.005)	.002	.032 (0.005)	.004
PrivacySetting*Stigma	—^d^	—	.0126 (2.14)	.06	—	—	.018 (.009)	.02
Title	.0158 (3.44)	.001	.0160 (3.48)	.006	.017 (.006)	<.001	.017 (.006)	<.001
Article	.148 (32.20)	<.001	.148 (32.20)	<.001	.222 (.058)	<.001	.222 (.058)	.001
Gift	.490 (77.11)	<.001	.490 (77.04)	<.001	.220 (.033)	<.001	.220 (.033)	<.001
Letter	.297 (46.16)	<.001	.297 (46.17)	<.001	.483 (.055)	<.001	.483 (.055)	<.001
Constant	1.91e-10 (.00)	>.99	1.33e-10 (.00)	.54	–.000 (.004)	.32	.000 (.004)	.83
*R* ^2^	.6081	N/A^e^	.6081	N/A	.552	N/A	.552	N/A

^a^intrctPHKS: interactive professional health care knowledge sharing.

^b^searchPHKS: searching professional health care knowledge sharing.

^c^Values in parentheses are the robust standard errors.

^d^Not available. Models 1 and 3 did not present the moderate effect of stigma.

^e^N/A: not applicable.

## Discussion

### Principal Findings

This study examined the role of a doctor’s privacy protection settings in OHCs on professional knowledge sharing. According to the data set from a distinguished OHC in China, both interactive professional health care knowledge sharing (intrctPHKS) and searching professional health care knowledge sharing (searchPHKS) are considered to be representative of a doctor’s professional health care knowledge sharing in OHCs. The stigma of diseases is estimated to moderate the association of privacy protection settings and professional health care knowledge sharing.

Our results indicate that our hypotheses on the effect of doctors’ privacy protection settings on professional health care knowledge sharing are significantly supported. The more privacy protection setting a doctor establishes, the more interactive their professional health care knowledge sharing will be. This means that if a doctor sets their interaction records as private, their patients will prefer to make multiple interactions, and more patients will consult this doctor. Thus, a higher performance of interactive professional health care knowledge sharing can be achieved. Similar studies in other privacy-sensitive contexts, such as in financial loans, e-commerce, and so on, have demonstrated that better privacy protection mechanisms increase user trust and, thus, increase user willingness to disclose personal information for better management [41,42].

However, a doctor’s privacy protection setting will limit the number of patients searching for professional health care knowledge online. This may lead to patients needing more time to find the right doctor, or they might not learn some useful information on the same health conditions. Therefore, doctors’ privacy protection settings restrict their searching for professional health care knowledge sharing with patients. Switching off privacy protection settings properly is good for increasing searching professional health care knowledge sharing. This result is also valid in financial fields, where the appropriate sharing of privacy can overcome the information asymmetry of financial credit [43]. However, it is more acceptable for OHC users to disclosure their online consultation records for searching professional health care knowledge sharing than for borrowers to disclose their financial records for overcoming the information asymmetry. This may be due to the nature of professional health care knowledge sharing, which is reciprocal, and disclosure behaviors can give patients a sense of achievement. However, financial privacy is related to personal property, which a person is usually unwilling to share [44]. The difference has also been confirmed by Xu et al [45] that privacy sensitivity varies across different types of websites (eg, in e-commerce, financial, and health care).

**Figure 1:**
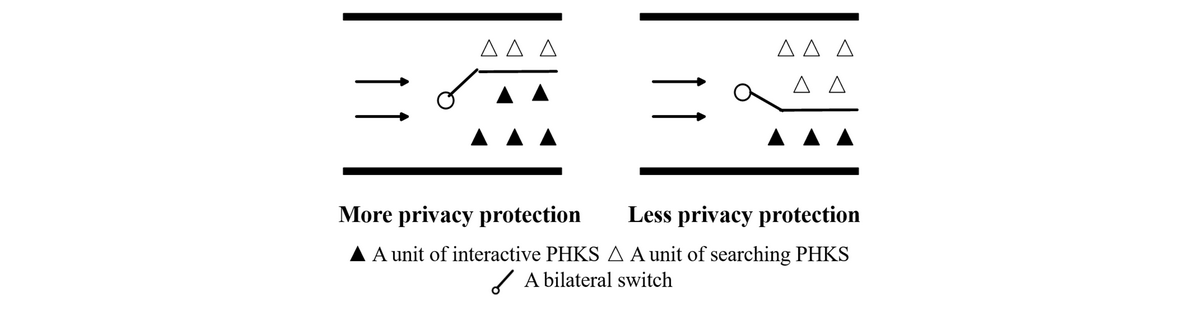
Illustration of the effect of privacy protection settings on interactive and searching professional health care knowledge sharing. PHKS: professional health care knowledge sharing.

As privacy protection settings positively affect interactive professional health care knowledge sharing and negatively affect searching professional health care knowledge sharing, it presents a bilateral switch of professional health care knowledge sharing, which is positive on one side and must at the same time be negative on the other side. The doctor who prefers to establish privacy protection will enjoy better performance in interactive professional health care knowledge sharing, but this weakens the performance of professional health care knowledge sharing searching. Conversely, a doctor who prefers public records will have better searching professional health care knowledge sharing and relatively weak interactive professional health care knowledge sharing. Figure 1 illustrates the metaphor of the bilateral switch. A doctor’s professional health care knowledge sharing is a process of professional health care knowledge flowing from doctors to patients, like blood flowing from one side to the other side in blood vessels. In Figure 1, the privacy protection setting is likened to a bilateral switch that is installed in the blood vessels to regulate blood shunting. When blood (professional health care knowledge sharing) meets the switch (privacy protection settings), a bifurcation moves upward (interactive professional health care knowledge sharing), and the other bifurcation flows downward (searching professional health care knowledge sharing). If a doctor creates a stronger privacy protection setting, the bilateral switch of professional health care knowledge sharing is a downward regulation, thus resulting in more of interactive professional health care knowledge sharing and less of searching professional health care knowledge sharing. However, when the doctor prefers weaker privacy protection settings, there will be more searching professional health care knowledge sharing and less interactive professional health care knowledge sharing, with the switch turning up.

**Figure 2:**
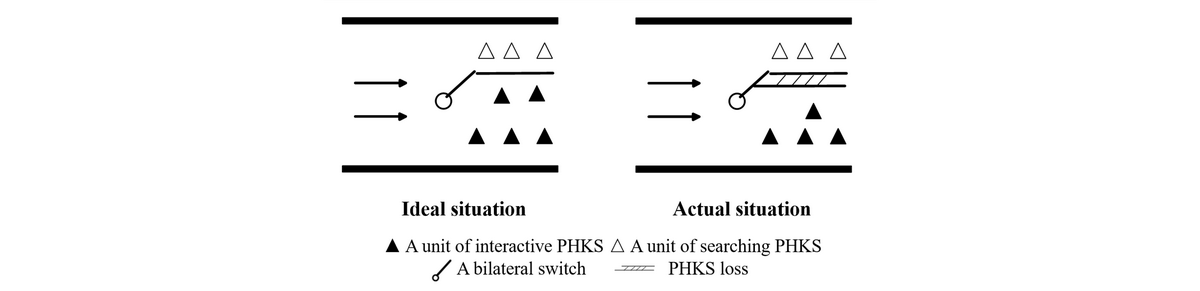
Illustration of the effect of privacy protection settings on interactive and searching professional health care knowledge sharing in an ideal and actual situation. PHKS: professional health care knowledge sharing.

However, as we noted previously, according to the Wald test, the absolute value of β_1_ in searching professional health care knowledge sharing is higher than that in interactive professional health care knowledge sharing. Therefore, when the same number of consultations are added that have a privacy protection setting, the loss of searching professional health care knowledge sharing is greater than that of the interactive professional health care knowledge sharing. In other words, the bilateral switch of professional health care knowledge sharing does not form a straight line as shown in Figure 1, but instead shows a thick switch that may cause a loss of professional health care knowledge sharing. We show an example in Figure 2.

**Figure 3:**
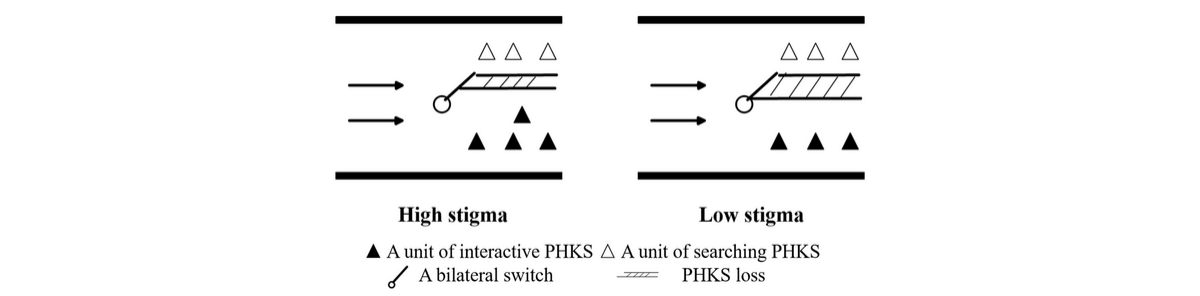
Illustration of the effect of disease stigma on interactive and searching professional health care knowledge sharing. PHKS: professional health care knowledge sharing.

In addition, empirical results indicate that the moderating effects of stigma positively affect the relationship between privacy protection settings and interactive professional health care knowledge sharing, and have a negative influence on the relationship between privacy protection settings and searching professional health care knowledge sharing. In other words, for high-stigma diseases, the increase in privacy protection settings is better for interactive professional health care knowledge sharing, while at the same time weakening the reduction of the searching professional health care knowledge sharing. This can be visualized as the stigma adjusting the thickness of the switch, namely professional health care knowledge sharing losses. The higher the stigma, the smaller the thickness of the switch (less loss). Conversely, the lower the stigma, the higher the thickness of the switch (more loss). This is because the virtual interaction mechanism of OHCs appears to be more acceptable by patients with high disease stigma. We show an example in Figure 3. OHCs recognize that they would have more visitors if they provide professional disease treatment support, as patients may disregard the disease stigma.

### Theoretical Implications

This study augments OHC-related research with several insights. First, the OHC, as a new application of a sharing economy, provides an efficient sharing platform for scarce professional health care knowledge [46]. Like in other forms of sharing economies, the opportunity for OHCs to share valuable professional health care knowledge is enormous. However, compared with other sharing economy applications, the OHC also faces privacy protection challenges due to the sensitivity and life-threatening nature of medical information. Although previous studies have studied privacy-protection technologies, patient privacy concerns, and intentions, the current OHC privacy protection mechanism is still incomplete. The privacy protection mechanism established by doctors was in an experimental stage in the target website of this study (Good Doctor). Privacy protection settings by doctors have the advantage that doctors can control whether to install privacy protection settings from a professional and objective perspective (compared with patients’ subjective perspectives). Therefore, we proposed a research framework that verifies the effect of a doctor’s privacy protection mechanism on professional health care knowledge sharing.

Second, we abstracted knowledge sharing in an OHC into interactive professional health care knowledge sharing and searching professional health care knowledge sharing, and empirically proved the significant differences in privacy protection settings between them. A privacy protection setting, like a bilateral switch of professional health care knowledge sharing, positively affects interactive professional health care knowledge sharing and negatively affects searching professional health care knowledge sharing. In addition to the positive effects of privacy protection settings, we uncovered the paradox between privacy protection settings and knowledge diffusion. In other words, the effects of privacy protection settings on knowledge sharing were not seen to be stable and required a balanced state of a bilateral switch. This is another paradox of privacy protection setting in addition to the privacy-personalization paradox [47], which can be denominated as the privacy-knowledge-sharing paradox.

We also examined the moderating effects of disease stigma on the association between privacy protection settings and doctors’ online professional health care knowledge sharing. Social stigma has been studied widely in the privacy field but rarely examined in existing studies on knowledge sharing. The empirical studies considered the direct or indirect influences of stigma on an individual’s intention to disclose information [21]. However, there is a lack of empirical studies that examine the direct or moderating effect of disease stigma on knowledge sharing. We determined that disease stigma has a positive moderating effect on the relationship of privacy protection settings with online professional health care knowledge sharing. In particular, this result indicates a facilitating effect of privacy protection settings on interactive professional health care knowledge sharing and an inhibiting effect of privacy protection settings on searching professional health care knowledge sharing.

### Practical Implications

This study provides several important practical implications for both OHC administrators and doctors. Our results indicate that a doctor’s privacy protection settings are the bilateral switch of professional health care knowledge sharing. For OHC managers, protecting patient privacy helps to improve the participation and continuous use intentions of patients. However, it also hinders the extensive spreading of professional health care knowledge, which can help users to realize the social value of the OHC and increase its influence. For doctors, their privacy protection settings help to reduce patient privacy concerns and thus encourage patients to provide more accurate information to provide better advice. In addition, the privacy protection setting renders the doctor’s professional health care knowledge more difficult for patients to access in a search, thus reducing the doctor’s “exposure” in the OHCs. The fewer patients the doctors have, the less knowledge they are likely to share. Therefore, we postulate that privacy protection settings are a bilateral switch of professional health care knowledge that needs balancing between OHC managers and doctors. The question is how to balance such a bilateral switch, which impacts the performance of both interactive professional health care knowledge sharing and searching professional health care knowledge sharing. Moreover, our empirical results improve the decision making of OHC managers and doctors. Specifically, the privacy protection setting has a cutoff effect on searching professional health care knowledge sharing and a promoting effect on interactive professional health care knowledge sharing. Furthermore, the cutoff effect is weaker than the promoting effect. Therefore, OHC managers and doctors can make their decision (by toggling the bilateral switch of professional health care knowledge sharing) according to whether their target is the OHC or the individual propensity. In addition, the moderating effect of disease stigma positively affects the relation between privacy protection settings and professional health care knowledge sharing. In other words, for a high-stigma disease, the professional health care knowledge sharing process is more friendly toward privacy protection settings. However, a low-stigma disease needs less privacy protection. This will help managers of OHCs and doctors to balance the bilateral switch of professional health care knowledge sharing based on disease stigma.

### Limitations and Future Research Directions

Our paper has some limitations that can serve as potential areas for future research. First, our research focuses on the OHC context. The methodologies and insights have the potential to be generalized to other wellness-care contexts, such as mHealth. However, examining the mHealth privacy paradox to compare the relationship and heterogeneity of the impact on patient behavior and outcomes under different health care contexts would be more beneficial and vital for future research.

Second, despite the collection of data from a typical OHC in China and the provision of a unique data set with doctors’ privacy protection settings, the general applicability of our conclusions may be limited. Future research could extend this study across different OHCs and cultural settings to enhance the generalizability of our study.

Additionally, although our results effectively explain the privacy protection settings of doctors on professional health care knowledge sharing in OHCs, in-depth interviews or surveys with patients could further investigate perceptions of patients on the relationship between privacy protection settings and professional health care knowledge sharing. Moreover, given the important role of patients in the online professional health care knowledge sharing process, in the future, researchers could also explore how patient-related variables (ie, health status, health conditions, disease severity, participant motivations and reasons) affect doctors’ online professional health care knowledge sharing with patients. Although both the difference-in-difference models (in our main analysis) and the cross-sectional models (in the robust check) showed significant verification of our hypotheses, they also indicate that the control variables may have strong explanatory power on doctors’ professional health care knowledge sharing (ie, gift 0.49, *P*<.001). These control variables may be the proxy variables for the quality of doctors’ treatment. Therefore, future research can deeply explore the effect of online response quality on professional health care knowledge sharing.

### Conclusion

The important role of OHCs in sharing health care knowledge has been widely acknowledged by both practitioners and researchers; however, significant gaps remain in our exploration of the privacy protection mechanism in OHCs. This study examined how doctors’ privacy protection setting choices affect their professional health care knowledge sharing in a Chinese OHC, a topic that, for the most part, has not been studied from the perspective of knowledge sharing. We used a fresh category of professional health care knowledge sharing; namely, interactive professional health care knowledge sharing and searching professional health care knowledge sharing. Doctors play different roles in these two kinds of professional health care knowledge sharing, both as active and passive actors, where different knowledge sharing processes occur. Using an empirical study on a large OHC, this study identified the bilateral role of privacy protection settings on professional health care knowledge sharing. It is a privacy-knowledge–sharing paradox in which the professional health care knowledge sharing process is not stable and calls for a balanced state. Our findings offer implications for doctors to gain a better understanding of how to set privacy protection for better professional health care knowledge sharing. Different privacy protection mechanisms can be suggested by OHC executives, which are customized to suit each level of disease stigma. We hope that this study promotes additional research to further enrich our understanding of professional health care knowledge sharing in OHCs.

## Appendix

Multimedia Appendix 1 Further previous research.

## Abbreviations

e-commerce: electronic commerce
OHC: online health community
