# Supplementary material for: Privacy Protection in Online Health Communities: Natural Experimental Empirical Study
Source: J Med Internet Res. 2020 May 21;22(5):e16246. doi: 10.2196/16246 (PMC7273234; doi:10.2196/16246)
Supplement: Multimedia Appendix 1 [file jmir_v22i5e16246_app1.doc]

## Appendix 1: Further Previous Research

#### Healthcare Knowledge Sharing

Knowledge sharing transpires when an individual, a team, and an organization share knowledge with other members of the governing body in the form of activities through various approaches [1]. Furthermore, it is an activity through which knowledge is exchanged between people, acquaintances, families, communities, or organizations [2].

The rapid rise of online communities has significantly stimulated interest in knowledge sharing in the last decade [3-5]. The two most representative forms of knowledge sharing online communities are user-generated websites and knowledge crowdsourcing. In user-generated websites (e.g., Wikipedia), knowledge is contributed by volunteers and embedded in the user-generated content [6]. In contrast, knowledge crowdsourcing is a platform for knowledge transactions (monetary knowledge sharing) based in online communities [7]. Most of the online health communities (OHCs) are reciprocal and categorized as user-generated websites, where professional healthcare knowledge sharing (PHKS) is embedded in the user-generated contents of doctors and patients.

PHKS in online communities can be shared among patients (e.g., in Wikipedia [8]), doctors (e.g., in physicians’ knowledge sharing in virtual communities [9, 10]), and also between doctors and patients. Doctors and patients differ in terms of status in PHKS contexts. The doctor, as the owner of a PHK domain, has more initiative when sharing it with patients. Indeed, PHKS between doctors and patients can be divided into interactive PHKS and searching PHKS. The interactive PHKS is embedded in doctor-patient communication, such as online and telephone consultations in OHCs. In fact, the searching PHKS is a process whereby patients search for and learn the user generated content in OHCs (e.g., doctors’ published articles, doctor-patient interactions in medical records, etc.), and as follows, learn and acquire doctors' PHK autonomously.

#### Privacy Protection

Users share uncountable quantities of personal healthcare information on the Internet. Openness in the online community, combined with the growing availability of technologies supporting the secondary use of personal healthcare information, can significantly increase the likelihood of the misuse of health data [11]. Personal healthcare information is a kind of privacy that refers to “the claim of individuals, groups, or institutions to determine for themselves when, how, and to what extent information about them is communicated to others” [12]. In order to better communicate with doctors or other patients in OHCs, patients have to upload personal healthcare information onto the Internet as much as possible. However, personal healthcare information in a digital format can be more easily copied, transmitted, and integrated. When it is revealed to unauthorized individuals and entities, they may misuse the data by stealing personal information to deceive friends and relatives, sending irritating advertisements, and by selling personal information to other businesses. Such disclosures may negatively affect individuals’ mental health [13], wealth [14], reputation, and even their healthy lives. The lack of user conﬁdence in online privacy has been identiﬁed as a major problem hampering the growth of an online community [15]. Li, Li [16] also verified that privacy issues could be regarded as perceived risks bundled with sharing knowledge in an OHC.

The implications of privacy protection in the online community have been both generally examined [17], as well as specifically, in the healthcare context [18]. Young and Quan-Haase [19] studied factors influencing the disclosure of health information on Facebook and the measures that people took to protect their privacy. Hartzler, Skeels [18] indicated that people often made errors in protecting their privacy when determining what health information was shared and with whom in their online community. Moreover, it has been proved that privacy protection mechanisms facilitate a patient’s participation in OHCs [11, 20]. Studies also applied emerging technologies, such as block chain, to protect the privacy of personal health information in OHCs [21-23].

However, privacy protection is also a double-edged sword [12]. In an OHC, the knowledge sharing process between doctors and patients requires patients to provide more personal healthcare information so that doctors can provide accurate diagnoses. Moreover, the more detailed personal health information shared, the more accurate will be the intelligent recommendation algorithms (for searching doctors, Intelligent triages) [24]. The development of OHCs based on technological innovation will largely depend on the information disclosure of users [25, 26]. Therefore, it is imperative to create a personalization-privacy tradeoff.

#### References

1. Bartol KM, Srivastava A. Encouraging knowledge sharing: the role of organizational reward systems. Journal of Leadership & Organizational Studies. 2002;9(1):64-76.

2. Hendriks P. Why share knowledge? The influence of ICT on the motivation for knowledge sharing. Knowledge and process management. 1999;6(2):91-100.

3. McLure Wasko M, Faraj S. “It is what one does”: why people participate and help others in electronic communities of practice. The Journal of Strategic Information Systems. 2000;9(2):155-73.

4. Bieber M, Engelbart D, Furuta R, Hiltz SR, Noll J, Preece J, et al. Toward virtual community knowledge evolution. Journal of Management Information Systems. 2002;18(4):11-35.

5. Kankanhalli A, Tan BC, Wei K-K. Contributing knowledge to electronic knowledge repositories: an empirical investigation. Mis Quarterly. 2005;29(1):113-43.

6. Ardichvili A, Page V, Wentling T. Motivation and Barriers to Participation in Virtual Knowledge-Sharing Communities of Practice. Journal of Knowledge Kanagement. 2003;7(1):64-77.

7. Haas MR, Hansen MT. Different knowledge, different benefits: toward a productivity perspective on knowledge sharing in organizations. Strategic Management Journal. 2007;28(11):1133-53.

8. Farič N, Potts HW. Motivations for Contributing to Health-Related Articles on Wikipedia: An Interview Study. J Med Internet Res. 2014;16(12):e260. PMID: 25498308. doi: 10.2196/jmir.3569.

9. Murad A, Hyde N, Chang S, Lederman R, Bosua R, Pirotta M, et al. Quantifying Use of a Health Virtual Community of Practice for General Practitioners’ Continuing Professional Development: A Novel Methodology and Pilot Evaluation. J Med Internet Res. 2019;21(11):e14545. PMID: 31774401. doi: 10.2196/14545.

10. Rolls K, Hansen M, Jackson D, Elliott D. How Health Care Professionals Use Social Media to Create Virtual Communities: An Integrative Review. J Med Internet Res. 2016;18(6):e166. PMID: 27328967. doi: 10.2196/jmir.5312.

11. De Choudhury M, Morris MR, White RW, editors. Seeking and sharing health information online: comparing search engines and social media. Proceedings of the 32nd annual ACM conference on Human factors in computing systems; 2014: ACM.

12. Westin AF. Privacy and freedom. Washington and Lee Law Review. 1968;25(1):166.

13. Kanter M, Afifi T, Robbins S. The impact of parents “friending” their young adult child on Facebook on perceptions of parental privacy invasions and parent–child relationship quality. Journal of Communication. 2012;62(5):900-17.

14. Walker K. The costs of privacy. Harv JL & Pub Pol'y. 2001;25:87.

15. Malhotra NK, Kim SS, Agarwal J. Internet Users' Information Privacy Concerns (IUIPC): the Construct, the Scale, and A Causal Model. Information Systems Research. 2004;15(4):336-55.

16. Li C, Li H, Suomi R. KNOWLEDGE SHARING IN A SMOKING CESSATION ONLINE COMMUNITY: A PRIVACY CALCULUS PERSPECTIVE. 2019.

17. Morris MR, Teevan J, Panovich K, editors. What do people ask their social networks, and why?: a survey study of status message q&a behavior. Proceedings of the SIGCHI conference on Human factors in computing systems; 2010: ACM.

18. Hartzler A, Skeels MM, Mukai M, Powell C, Klasnja P, Pratt W, editors. Sharing is caring, but not error free: Transparency of granular controls for sharing personal health information in social networks. AMIA Annual Symposium Proceedings; 2011: American Medical Informatics Association.

19. Young AL, Quan-Haase A, editors. Information revelation and internet privacy concerns on social network sites: a case study of facebook. Proceedings of the fourth international conference on Communities and technologies; 2009: ACM.

20. Zhou L, Bao J, Watzlaf V, Parmanto B. Barriers to and Facilitators of the Use of Mobile Health Apps From a Security Perspective: Mixed-Methods Study. JMIR Mhealth Uhealth. 2019;7(4):e11223. PMID: 30990458. doi: 10.2196/11223.

21. Zhu X, Shi J, Lu C. Cloud Health Resource Sharing Based on Consensus-Oriented Blockchain Technology: Case Study on a Breast Tumor Diagnosis Service. J Med Internet Res. 2019;21(7):e13767. PMID: 31339106. doi: 10.2196/13767.

22. Wang Y, Sun L, Hou J. Hierarchical Medical System Based on Big Data and Mobile Internet: A New Strategic Choice in Health Care. JMIR Med Inform. 2017;5(3):e22. PMID: 28790024. doi: 10.2196/medinform.6799.

23. Bender JL, Cyr AB, Arbuckle L, Ferris LE. Ethics and Privacy Implications of Using the Internet and Social Media to Recruit Participants for Health Research: A Privacy-by-Design Framework for Online Recruitment. J Med Internet Res. 2017;19(4):e104. PMID: 28385682. doi: 10.2196/jmir.7029.

24. Seppälä A, Nykänen P, Ruotsalainen P. Privacy-Related Context Information for Ubiquitous Health. JMIR Mhealth Uhealth. 2014;2(1):e12. PMID: 25100084. doi: 10.2196/mhealth.3123.

25. Zhang X, Liu S, Chen X, Wang L, Gao B, Zhu Q. Health information privacy concerns, antecedents, and information disclosure intention in online health communities. Information & Management. 2018;55(4):482-93.

26. Deng Z, Hong Z, Ren C, Zhang W, Xiang F. What Predicts Patients’ Adoption Intention Toward mHealth Services in China: Empirical Study. JMIR Mhealth Uhealth. 2018;6(8):e172. PMID: 30158101. doi: 10.2196/mhealth.9316.
